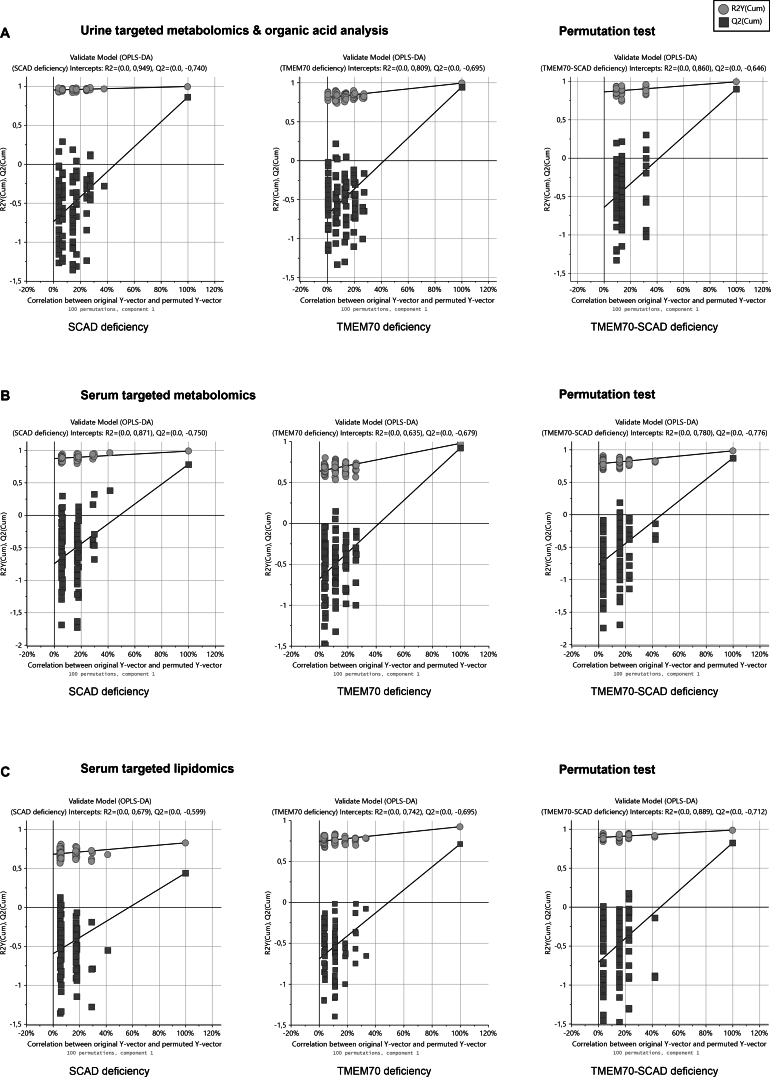# Comprehensive metabolomic/lipidomic characterization of patients with mitochondrial ATP synthase, short-chain acyl-CoA dehydrogenase and combined variant deficiencies

**DOI:** 10.1016/j.heliyon.2025.e42797

**Published:** 2025-02-21

**Authors:** Dana Dobešová, Matúš Prídavok, Radana Brumarová, Aleš Kvasnička, Barbora Piskláková, Eliška Ivanovová, Katarína Brennerová, Jana Šaligová, Ľudmila Potočňáková, Simona Drobňaková, Jana Potočňáková, David Friedecký

**Affiliations:** a Laboratory for Inherited Metabolic Disorders, Faculty of Medicine and Dentistry, Palacký University Olomouc, Olomouc, Czech Republic; b Department of Laboratory Medicine, Centre for Inherited Metabolic Disorders, National Institute of Children's Diseases, Bratislava, Slovak Republic; c Department of Clinical Biochemistry, University Hospital Olomouc, Czech Republic; d Department of Pediatrics, Centre for Inherited Metabolic Disorders, National Institute of Children's Diseases, Bratislava, Slovak Republic; e Metabolic Outpatient Clinic, Children's Faculty Hospital, Košice, Slovak Republic; f Department of Pediatric and Adolescent Medicine, Faculty of Medicine, Pavol Josef Safarik University, Košice, Slovak Republic; g Second Faculty of Medicine, Charles University, Praha, Czech Republic

**Keywords:** Mitochondrial disorders, Oxidative phosphorylation, TMEM70, SCAD, Metabolomics, Lipidomics

## Abstract

**Objective:**

This study aims to characterize the metabolic alterations in patients with inherited mitochondrial enzymopathies. We focused on wide-coverage targeted metabolomic, organic acid and lipidomic analyses of patients with TMEM70 deficiency (TMEM70d), short-chain acyl-CoA dehydrogenase deficiency (SCADd), and individuals with both deficiencies (TMEM70d-SCADd).

**Methods:**

Serum and urine samples were collected from patients with TMEM70d (n = 13), SCADd (n = 11), TMEM70d-SCADd (n = 3), and controls (n = 38). Analyses were conducted using high-performance liquid chromatography coupled with tandem mass spectrometry (LC-MS/MS). Univariate and multivariate statistical evaluation was performed to identify significant metabolic differences between patient groups and controls.

**Results:**

Distinct metabolic profiles were observed in urine and serum samples of patients with TMEM70d, SCADd, and TMEM70d-SCADd compared to controls. Urinary metabolomics revealed significant elevations in butyrylcarnitine and metabolites related to branched-chain amino acid degradation in SCADd and TMEM70d-SCADd patients. Serum metabolomic analysis indicated alterations in pyruvate metabolism, citric acid cycle intermediates, and acylcarnitine metabolism in TMEM70d and TMEM70d-SCADd patients. Lipidomic analysis showed decreased levels of glycerophospholipids and sphingolipids across all patient groups.

**Conclusion:**

Patients with TMEM70d, SCADd, and TMEM70d-SCADd exhibit distinct metabolic signatures characterized by disturbances in energy metabolism, amino acid degradation, and lipid homeostasis. The combination of TMEM70d and SCADd leads to synergistic metabolic effects, emphasizing the importance of comprehensive metabolic profiling in understanding complex mitochondrial disorders and identifying potential biomarkers for diagnosis and treatment monitoring.

## Highlights

• Metabolomic and lipidomic analyses serve as an important tool to assess pathobiochemical changes in mitochondrial enzymopathies.
• Distinct imbalances in the citric acid cycle, degradation of branched-chain amino acids and acylcarnitine metabolism were revealed in patients with mitochondrial disorders.
• Marked lipid dysregulation in glycerophospholipids and sphingolipids levels was observed in all studied patients
• The combination of TMEM70d and SCADd leads to a synergistic metabolic effect in patients with these mutations.

## Graphical abstract

**Figure:**
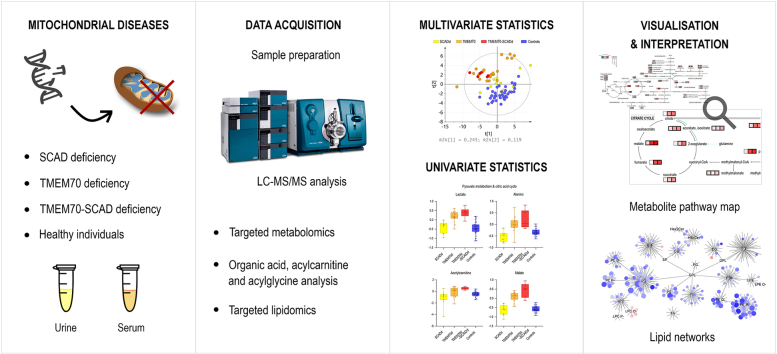

**Table:** Abbreviations

2MGC	2-Methylglutaconate
3MG	3-Methylglutarate
3MGC	3-Methylglutaconate
ADP	Adenosine diphosphate
ATP	Adenosine triphosphate
Cer	Ceramides
CON	Control group
DG	Diacylglycerols
DHAP	Dihydroxyacetone phosphate
EMA	Ethylmalonate
FA	Free fatty acids
FAD(H_2_)	Flavin adenine dinucleotide
G3P	Glyceraldehyde-3-phosphate
GL	Glycerides
GPL	Glycerophospholipids
Hex2Cer	Dihexanoylceramides
HexCer	Hexanoylceramides
IMDs	Inherited metabolic disorders
LC-MS/MS	Liquid chromatography coupled to tandem mass spectrometry
LPC	Lysophosphatidylcholines
LPC P-/O-	Lysophosphatidylcholine plasmalogens/plasmanyls
LPE	Lysophosphatidylethanolamines
LPE O-	Lysophosphatidylethanolamine plasmanyls
MELAS	Mitochondrial encephalomyopathy, lactic acidosis, and stroke-like episodes syndrome
MSA	Methylsuccinate
NAD(H)	Nicotineamide adenine dinucleotide
NARP	Neurogenic weakness, ataxia and retinitis pigmentosa
NO	Nitric oxide
OPLS-DA	Orthogonal partial least square discriminant analysis
OXPHOS	Oxidative phosphorylation
PC	Phosphatidylcholines
PC P-/O-	Phosphatidylcholine plasmalogens/plasmanyls
PCA	Principal component analysis
PE	Phosphatidylethanolamines
PE P-/O-	Phosphatidylethanolamine plasmalogens/plasmanyls
PG	Phosphatidylglycerols
PI	Phosphatidylinositols
PS	Phosphatidylserines
SCAD	Short-chain acyl-coenzyme A dehydrogenase
SCADd	Short-chain acyl-coenzyme A dehydrogenase deficiency
SM	Sphingomyelins
SP	Sphingolipids
TG	Triacylglycerols
TMEM70	Transmembrane protein 70
TMEM70d	Transmembrane protein 70 deficiency
TMEM70d-SCADd	Combined defect of SCAD and TMEM70

## 1 Introduction

Mitochondria, as a part of all nucleated human cells, play a central role in various metabolic pathways such as β-oxidation, citric acid cycle, urea cycle, ketone bodies metabolism and degradation of branched-chain amino acids. One of the main functions is to obtain energy in the form of adenosine triphosphate (ATP) by oxidative phosphorylation (OXPHOS). The OXPHOS system comprises five multimeric complexes embedded in the inner mitochondrial membrane. Three of them (complexes I, III, and IV) are responsible for the transport of protons from the matrix into the intermembrane space. ATP synthase (complex V, EC 7.1.2.2) uses this electrochemical proton gradient to produce ATP from adenosine diphosphate (ADP) and inorganic phosphate by a rotary mechanism. Human ATP synthase consists of 29 subunits and requires transmembrane protein 70 (TMEM70), which assembles the c8-ring of the Fo-domain, for its proper function [1,2]. A significant reduction in fully assembled ATP synthase and its activity (70–80 %) is characteristic of TMEM70 deficiency (TMEM70d, OMIM #614052), a rare autosomal recessive mitochondrial disorder [3,4].

The *TMEM70* gene is located on chromosome 8 and contains three exons. Pathological variants of *TMEM70* were first discovered in 2008 in the Czech Republic [5]. The most common mutation c.317-2A > G leads to abnormal splicing and loss of *TMEM70* transcript [6,7]. In total, over 20 different mutations have been described [8]. By the end of 2014, more than 50 cases of TMEM70d worldwide were identified [6]. Patients suffer from a variety of clinical problems like as lactic acidosis, hyperammonemia, hyperuricemia, 3-methylglutaconic aciduria, prematurity, psychomotor delay, encephalo-cardiomyopathy, persistent pulmonary hypertension, microcephaly, facial dysmorphisms, hypotonia and cryptorchidism [9].

Short-chain acyl-CoA dehydrogenase (SCAD, EC 1.3.8.1) deficiency (SCADd; OMIM #201470), another mitochondrial disease, is a rare autosomal recessive fatty acid β-oxidation disorder. The cause is mutations in the *ACADS* gene, which is located on chromosome 12q24.31 and encodes the flavin adenine dinucleotide (FAD)-dependent enzyme SCAD [10]. The major substrate for SCAD is butyryl-CoA, formed during the β-oxidation spiral when fatty acid chains are shortened. As a consequence of SCAD deficiency, butyryl-CoA accumulates in the form of conjugates - butyrylcarnitine and butyrylglycine in mitochondria and partially in the cytosol [11]. Butyrylcarnitine, a major marker of SCADd, could be revealed by screening for acylcarnitines in the dry blood spot in the Newborn screening (NBS) program [11,12]. Confirmation of the diagnosis involves finding ethylmalonate (EMA) and methylsuccinate (MSA), which also arise from excess butyryl-CoA, in the urinary organic acid analysis [13].

The global incidence of SCADd ranges from 1:35,000 to 1:50,000 [14]. However, according to research from 2016, the prevalence in Slovakia was 1:10,000 in Caucasian newborns and 1:100 in the Roma population [15]. There are approximately 70 mutations, and the most common variants include c.511C > T and c.625G > A [16]. Patients with SCADd are usually asymptomatic and there is a weak association between genotype and phenotype. Therefore, several countries have removed SCADd from their NBS program [17,18]. However, cases of serious threats to life have also been described [19]. Clinical characteristics may include developmental delay, hypotonia, epilepsy, hypoglycemia, dysmorphic facial features, seizures, vomiting, failure to thrive, hepatic dysfunction and metabolic acidosis [14,20].

This study aimed to characterize the overall pathobiochemical metabolic changes in patients with TMEM70d, SCADd and especially in individuals with a combination of both deficiencies. A targeted metabolomic and lipidomic approach was used for serum examination, and urine assessment was performed by targeted metabolomics in conjunction with organic acids, acylcarnitines and acylglycines analysis (from now on referred to as organic acid analysis) [21,22]. All three methods are based on high-performance liquid chromatography coupled with tandem mass spectrometry (LC-MS/MS). This work may contribute to the understanding of the processes associated with these disorders and to the identification of new potential biomarkers.

## 2 Material and methods

### 2.1 Chemicals and reagents

Methanol (MeOH), acetonitrile (ACN), isopropanol (IPA), water, acetic acid, ammonium hydroxide, ammonium acetate and ammonium formate (all LC-MS grade) were purchased from Sigma-Aldrich (St. Louis, MO, USA). SPLASH® LIPIDOMIX® Mass Spec Standard mixture and labeled ceramide (d18:1-d_7_/15:0) were obtained from Avanti Polar Lipids (Alabaster, AL, USA). Labeled arachidonic acid-d_8_ was acquired from the Cayman Chemical Company (Ann Arbor, MI, USA). For targeted metabolomics and organic acid analysis, the internal standards such as uracil-^15^N_2_ and butyryl-L-carnitine-(N-methyl-d_3_) hydrochloride, L-leucine-5,5,5-d_3_, adipic acid-d_10_, were obtained from Sigma Aldrich (St. Louis, MO, USA). Creatinine-d_3_ (methyl-d_3_) was acquired from CDN isotopes (Pointe-Claire, QC, Canada).

### 2.2 Sample collection

Serum and urine samples were collected from 11 SCADd patients (11× serum, 13× urine), 13 TMEM70d patients (21× serum, 25× urine), 3 TMEM70d-SCADd individuals (6× serum, 5× urine) and 38 controls (38× serum, 37× urine) in collaboration with the Centre for Inherited Metabolic Disorders, National Institute of Children's Diseases in Bratislava and Metabolic clinic, Children's Faculty Hospital in Košice. Clinical information (the first symptoms began to appear after birth), type of mutation, treatment, diet and number of samples are depicted in Table 1. Written informed consent to participation in the study was obtained from all patients before sampling. The study was approved by the Ethics Committee of The National Institute of Children's Diseases, Bratislava, Slovakia (No. EK6/3/2023). The collected samples were frozen and stored in 1.0 ml tubes at −80 °C before sample processing and analysis.

**Table 1:** Information about the patients with SCADd, TMEM70d and TMEM70d-SCADd included in the study. Median age for male/female groups: 2y7m/1y10m - SCADd, 3y3m/3y4m - TMEM70d, 2y5m/1y10m - TMEM70d-SCADd, 4y8m/5y8m – Controls). Number of male/female (23/15) in the control group.

Diagnosis	Sex	Serum sampling (age)	Urine sampling (age)	Mutation	Clinical information	Diet	Treatment
TMEM70d	Female	S1 (2y1m) S2 (2y2m) S3 (2y4m)	U1 (2y1m) U2 (2y2m) U3 (2y4m)	c.317-2A > G	psychomotor retardation, growth retardation, hypotonia, hypertrophic cardiomyopathy	limited carbohydrate intake, increased fat intake	thiamine, NaHCO_3_, MCT oil, L-carnitine
TMEM70d	Male	S1 (2y6m)	–	c.317-2A > G	psychomotor retardation, growth retardation, hypotonia, hypotrophy, hypertrophic cardiomyopathy	limited carbohydrate intake, increased fat intake	thiamine
TMEM70d	Female	S1 (2y1m)	U1 (1y4m) U2 (1y8m) U3 (2y1m) U4 (2y4m) U5 (2y7m)	c.317-2A > G	psychomotor retardation, growth retardation, hypotonia, hypotrophy, hypertrophic cardiomyopathy	limited carbohydrate intake, increased fat intake	thiamine, NaHCO_3_, MCT oil, L-carnitine
TMEM70d	Female	S1 (6y) S2 (6y6m) S3 (7y6m)	U1 (6y)	c.317-2A > G	psychomotor retardation, growth retardation, hypotonia, hypotrophy, stigmatization	limited carbohydrate intake, increased fat intake	thiamine, NaHCO_3_, MCT oil, L-carnitine
TMEM70d	Male	S1 (12y6m) S2 (13y1m)	U1 (12y6m) U2 (13y1m)	c.317-2A > G	psychomotor retardation, growth retardation, hypotonia, hypotrophy, stigmatization, strabismus	limited carbohydrate intake, increased fat intake	thiamine, NaHCO_3_, MCT oil, L-carnitine
TMEM70d	Male	S1 (1y5m) S2 (3y) S3 (3y3m) S4 (3y3m)	U1 (1y5m) U2 (3y)	c.317-2A > G	psychomotor retardation, growth retardation, hypotonia, hypotrophy, hypertrophic cardiomyopathy	limited carbohydrate intake, increased fat intake	thiamine, NaHCO_3_, MCT oil, L-carnitine
TMEM70d	Female	S1 (1y)	U1 (1y)	c.317-2A > G	growth retardation, hypotonia, hypotrophy, stigmatization	limited carbohydrate intake, increased fat intake	thiamine, NaHCO_3_, MCT oil, L-carnitine
TMEM70d	Female	S1 (2y3m)	–	c.317-2A > G	psychomotor retardation, growth retardation, hypotonia, hypotrophy, stigmatization	limited carbohydrate intake, increased fat intake	thiamine, NaHCO_3_, MCT oil, L-carnitine
TMEM70d	Female	–	U1 (3y7m)	c.317-2A > G	microcephaly, growth retardation, hypotrophy, recurrent pneumonia	normal diet	thiamine
TMEM70d	Female	S1 (6y2m) S2 (6y4m)	U1 (3y8m) U2 (3y8m) U3 (4y6m) U4 (4y7m) U5 (4y8m)	c.317-2A > G	psychomotor retardation, growth retardation, hypotonia, hypertrophic cardiomyopathy, epilepsy	no additional information	arginine, L-carnitine, biogaia, thiamine, enap, coenzyme Q10, riboflavin, MCT oil, pyridoxine
TMEM70d	Female	S1 (16y7m) S2 (17y11m)	U1 (16y7m)	c.317-2A > G	psychomotor retardation, growth retardation, microcephaly, hypertrophic cardiomyopathy, dwarfism, strabismus	increased fat intake; low protein diet during metabolic crisis	verospiron, enalapril, L-carnitine, vitamin D, B-complex
TMEM70d	Male	–	U1 (5y7m) U2 (6y4m)	c.317-2A > G	psychomotor retardation, growth retardation, hypotonia, hypotrophy, hypertrophic cardiomyopathy, microcephaly, micrognathia	increased fat intake; low protein diet during metabolic crisis, maltodextrin	NaHCO_3_, thiamine, B-complex, L-carnitine, vigantol, acidum folicum, cebion, calcium, enap
TMEM70d	Male	S1 (2y5m)	U1 (3y3m) U2 (3y5m)	c.317-2A > G	psychomotor retardation, growth retardation, hypotonia, hypotrophy, testicular retention	limited carbohydrate intake, increased fat intake	thiamine, NaHCO_3_, MCT oil, L-carnitine
TMEM70d-SCADd	Female	S1 (1y) S2 (1y2m)	U1 (1y) U2 (1y2m)	c.317-2A > G + c.625G > A	psychomotor retardation, growth retardation, hypotonia, hypotrophy, polydactylism, hypertrophic cardiomyopathy, strabismus	nutrini pepisorb	thiamine, MCT oil, maltofer, ursosan, duphalac, Ac.folicum
TMEM70d-SCADd	Female	S1 (2y5m) S2 (3y2m) S3 (3y2m)	U1 (1y1m)	c.317-2A > G + c.625G > A, c.310_312delGAG	psychomotor retardation, growth retardation, microcephaly, hypertrophic cardiomyopathy, strabismus, nephropathy	moderately increased fat intake; low protein diet during metabolic crisis	thiamine, pyridoxine, L-carnitine, B-complex, Sholov solution without calcium, arginine
TMEM70d-SCADd	Male	S1 (8y)	U1 (6y9m) U2 (8y)	c.317-2A > G + c.310_312delGAG	psychomotor retardation, growth retardation, hypotonia, hypertrophic cardiomyopathy, strabismus	moderately increased fat intake; low protein diet during metabolic crisis	NaHCO_3_, thiamine, pyridoxine, vitamin B12, vitamin E, coenzyme Q10, thiapridal, prothazine, L-carnitine
SCADd	Female	S1 (1y1m)	U1 (1y1m)	c.310_312delGAG, c.1138C > T	asymptomatic	normal diet; avoid starvation and hypoglycemia; polysaccharides at night	maltodextrin
SCADd	Male	S1 (1y9m) S2 (3y3m)	U1 (1y2m) U2 (3y3m)	c.1138C > T	asymptomatic, arthrogryposis	normal diet; avoid starvation and hypoglycemia; polysaccharides at night	maltodextrin
SCADd	Male	S1 (5y11m) S2 (6y3m)	U1 (5y7m) U2 (5y11m)	c.310_312delGAG	cortical atrophy, psychomotor retardation, microcephaly, immunodeficiency	normal diet; avoid starvation and hypoglycemia; polysaccharides at night	maltodextrin
SCADd	Male	S1 (2y3m)	U1 (2y3m) U2 (2y7m)	c.310_312delGAG	asymptomatic	normal diet; avoid starvation and hypoglycemia; polysaccharides at night	maltodextrin
SCADd	Female	–	U1 (1y10m)	c.625G > A, c.1138C > T	asymptomatic	normal diet; avoid starvation and hypoglycemia; polysaccharides at night	maltodextrin
SCADd	Male	S1 (1y10m)	–	c.310_312delGAG	asymptomatic	normal diet; avoid starvation and hypoglycemia; polysaccharides at night	maltodextrin
SCADd	Male	–	U1 (1y6m)	c.1138C > T	asymptomatic	normal diet, avoid starvation, and hypoglycemia, polysaccharides at night	maltodextrin
SCADd	Male	S1 (2y11m)	U1 (2y11m)	c.310_312delGAG	asymptomatic	normal diet; avoid starvation and hypoglycemia; polysaccharides at night	maltodextrin
SCADd	Male	S1 (3y3m)	U1 (3y3m)	c.310_312delGAG	asymptomatic, language delay	normal diet	untreated
SCADd	Male	S1 (2y7m)	–	c.310_312delGAG	asymptomatic	normal diet	untreated
SCADd	Female	S1 (3y5m)	U1 (1y1m) U2 (2y4m)	c.310_312delGAG	asymptomatic	normal diet	untreated

### 2.3 Clinical characteristics of patients with TMEM70d-SCADd

TMEM70d-SCADd patients manifested clinically very similarly to TMEM70d individuals. In most cases, they were born prematurely and were observed to have hypotonia, microcephaly, hypertrophic cardiomyopathy, and persistent pulmonary hypertension. They developed growth and psychomotor retardation. Some patients presented with strabismus. Basic laboratory findings showed metabolic acidosis, hyperammonemia and hyperlactatemia. Urinary organic acid analysis revealed 3-methylglutaconic and ethylmalonic aciduria.

### 2.4 Sample preparation

Serum for targeted metabolomic and lipidomic analysis was prepared separately. Randomized samples were transferred from −80 °C to −20 °C overnight and thawed on ice the following day. An aliquot of serum (5 μl) was taken from each sample to form a quality control (QC) sample, which was then treated as the other samples. Samples were vortexed for 10 s, metabolites and lipids were extracted by mixing 25 μl of serum with 75 μl of extraction solution containing the stable labeled internal standards and MeOH for metabolomics (5 μM creatinine-d_3_ (methyl-d_3_), L-leucine-5,5,5-d_3_, butyryl-L-carnitine-(N-methyl-d_3_) hydrochloride, 10 μM uracil-^15^N_2_ and adipic acid-d_10_) or IPA for lipidomics (5 % SPLASH® LIPIDOMIX® Mass Spec Standard mixture, 1 μM ceramide d18:1-d_7_/15:0 and 10 μM arachidonic acid-d_8_), and then samples were stored overnight at −80 °C for deproteinization. The following day, the mixtures were centrifuged (20 min, 15,000×*g*, 4 °C) and the supernatants were transferred into glass LC-MS vials. Samples were again randomized and subjected to analysis, separately. QC aliquots were measured at every sixth injection and were used to monitor instrument stability and during data processing.

Urine samples were diluted to a creatinine concentration of 1 mmol/L and prepared by the same extraction procedure as serum using an extraction mix with a modified ratio (urine/MeOH with internal standards, 50/50, v/v). The QC sample was prepared by pooling 5 μl of each urine sample after normalization to creatinine concentration and used with the same intention as for the serum analysis. Urine samples were analyzed by targeted metabolomics and wide coverage LC/MS-MS analysis of organic acids.

### 2.5 Methods

**Fig. 1:**
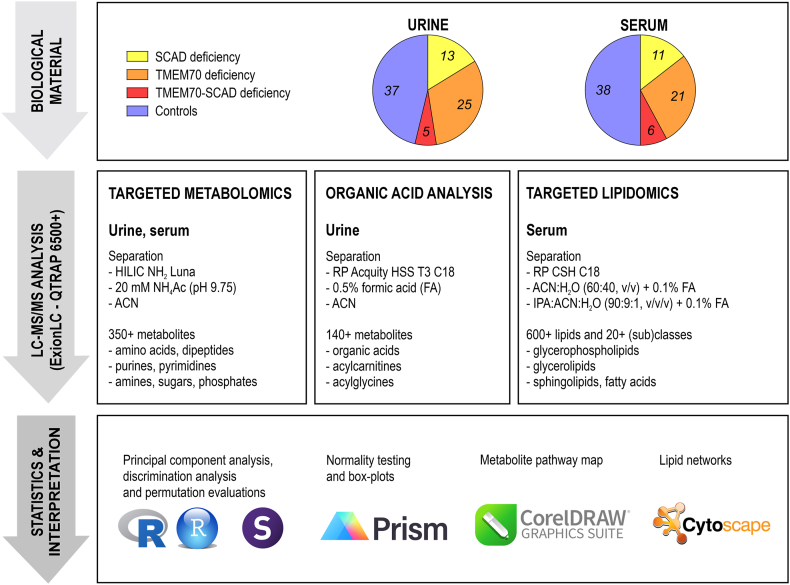
Summary of the metabolomic-lipidomic workflow of this study.

Wide-coverage targeted metabolomics, lipidomics, and organic acid analysis (Fig. 1) were carried out by an LC-MS/MS system ExionLC™ with QTRAP® 6500+ (Sciex, Framingham, MA, USA) equipped with electrospray ionization. All methods utilized simultaneous analysis in both positive/negative modes with polarity switching using the scheduled multiple reaction monitoring. Detailed information about the targeted metabolomic [23], lipidomic [24] and organic acid analysis [21] methods, chromatography and mass spectrometry settings are provided in our previous published works and in Supplementary Table S1.

To verify the linearity of the measurements and to support the ability of the methods to evaluate relative changes in analyte abundance between samples, an injection experiment was performed for each approach (metabolomics, lipidomics, organic acids) using a QC sample in injection range of 0.25–1.00 μl (4 point calibration) and 0.50–3.00 μl (6 point calibration) for metabolomics/organic acids and lipidomics, respectively. A linear response R^2^ > 0.9 was achieved for 92.2 % analytes within urine metabolomics, 96.9 % analytes in urine organic acid analysis, 97.6 % analytes in serum metabolomics and 94.1 % within lipidomics (Supplementary Table S1).

### 2.6 Data processing and statistical analysis

Analyst (v1.7.1) and SCIEX OS (v2.0) software (Sciex, Framingham, MA, USA) were used to acquire the raw data from the mass spectrometer. For wide-coverage metabolomic and lipidomic experiments, relative quantification based on analyte area ratios relative to internal standards was applied. The raw data and datasets generated and/or analyzed in this study are available in the MassIVE repository (ID MSV000094418) and at https://doi.org/doi:10.25345/C5D50G81T.

The data were processed and statistically evaluated using the Metabol package [25] in R software (v4.0.3, www.r-project.org, 2019). Local estimated smoothing signal (LOESS) correction was applied to each dataset separately based on the QC samples. Metabolites and lipids with the coefficient of variation (CV) higher than 30 % for the QC samples were excluded. Serum metabolomic and lipidomic datasets were evaluated separately, but urinary metabolomics was combined with the wide coverage organic acids LC-MS/MS analysis before statistical evaluation.

For multivariate statistical analysis, mean centering and natural logarithm (ln) transformation were applied to the data. Unsupervised principal component analysis (PCA), followed by supervised orthogonal partial least square discriminant analysis (OPLS-DA) were performed on the final datasets in SIMCA software (v17, Umetrics, Umeå, Sweden). The accuracy and predictive ability of the models were verified using permutation tests (n = 100). The normal distribution of the data was tested using the Shapiro-Wilk test (Supplementary Table S2) in GraphPad Prism (v10.0, GraphPad Software, San Diego, CA, USA). Based on its result, the non-parametric Mann–Whitney *U* test was applied. Benjamini-Hochberg correction with false discovery ratio = 0.05 was utilized to reduce false positive results. To evaluate clinical effectiveness (AUC, sensitivity and specificity), ROC analysis of all statistically significant metabolites and lipid was performed in SPSS version 27 (Supplementary Table S4). Visualisation of univariate outputs (p-value and fold change) was performed using box plots for metabolites (GraphPad Prism) and networks (Cytoscape, v3.10.0, Bethesda, MD, USA [26]) for lipids. The metabolite pathway map was constructed based on the fold changes of metabolites obtained by targeted metabolomics and wide coverage organic acid LC/MS-MS analyses (CorelDRAW, v20, Ottawa, Ontario, Canada). Lipid networks were constructed to visualise systematic changes in lipid classes and subclasses, where the node sizes correspond to -log p-values and red/blue colors correspond to ln fold change values. The power of study (Cohen's D value) was calculated at 1.37 and 0.74 for 5 and 25 urine samples from patients with TMEM70d-SCADd and TMEM70d compared to 37 healthy controls (for independent *t*-test) in Statistica software (TIBCO Statistica, v14, Palo Alto, CA, USA).

## 3 Results

### 3.1 Urine targeted metabolomics and organic acid analysis

**Fig. 2:**
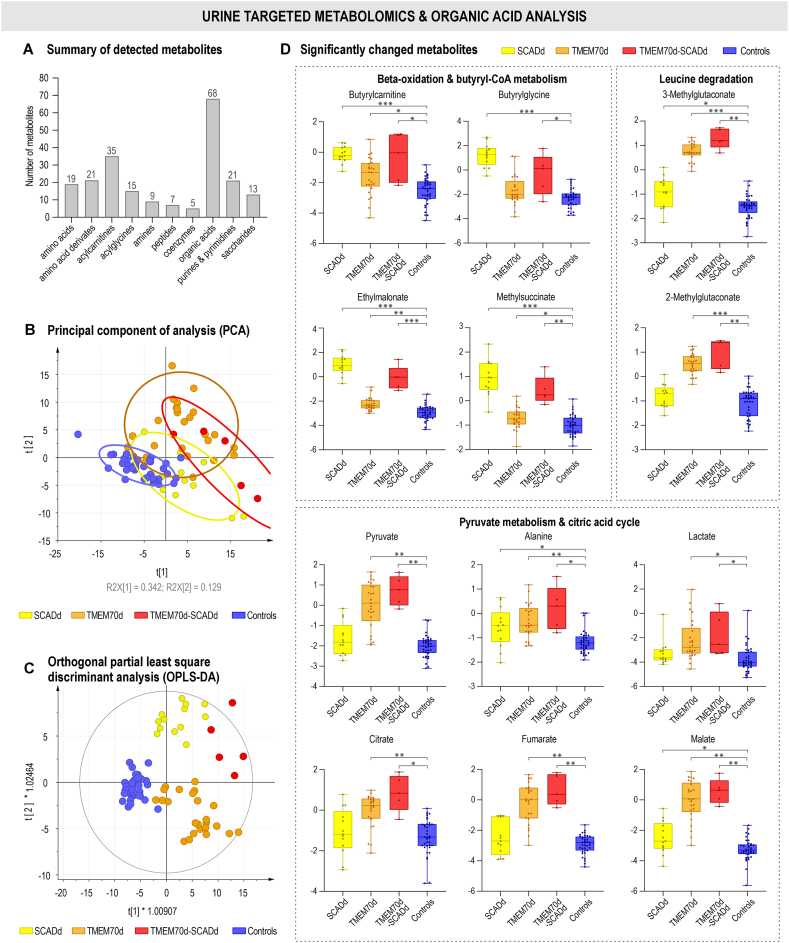
Urine metabolomics & organic acid analysis. The number of metabolites detected within groups in urine samples (A). Multivariate statistical analysis (B and C) unsupervised PCA and supervised OPLS-DA. Colour-coded sample groups: SCADd (yellow), TMEM70d (orange) and TMEM70d-SCADd (red) patients and control subjects (blue). Summary of the most significantly changed ones (D). Statistical significance was assessed by p-values after Benjamini-Hochberg correction (∗<0.05, ∗∗<0.01, ∗∗∗<0.001), the y-axis represents the relative abundance after the application of internal standards, LOESS correction and ln transformation.

The combination of targeted urinary metabolomics and organic acid analysis detected 213 metabolites (Fig. 2A) belonging to amino acids, amino acid derivatives, acylcarnitines, acylglycines, amines, peptides, coenzymes, organic acids, purines and pyrimidines, and carbohydrates. Unsupervised PCA showed separation of patient groups from controls with 47.1 % of variance explained. OPLS-DA further distinguished SCADd, TMEM70d and TMEM70d-SCADd, with TMEM70d-SCADd grouping closer to SCADd (Fig. 2B and C). The robustness of the OPLS-DA model was assessed using permutation calculation, where statistically significant accuracies of the model (R2Y = 0.995/0.993/0.992) and predictive abilities (Q2 = 0.859/0.943/0.897) values for SCADd/TMEM70d/TMEM70d-SCADd vs. control group were achieved, respectively (Supplementary Fig. S1A).

**Fig. 3:**
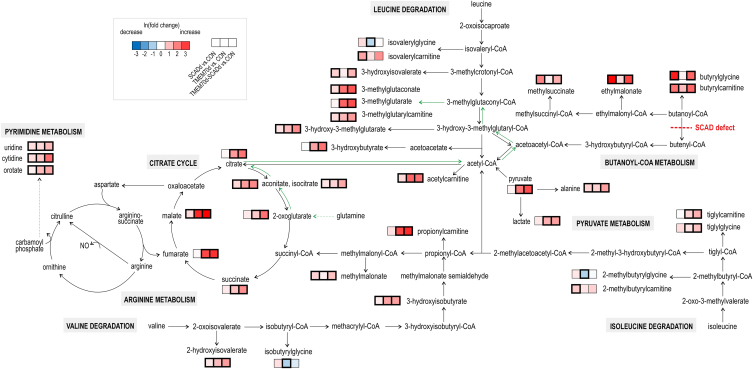
Metabolic map summarizing alterations in urinary metabolites in SCADd, TMEM70d and TMEM70d-SCADd patients compared to controls (CON). Significant changes in metabolites after Benjamini-Hochberg correction are highlighted by bold border. The used data were treated with internal standards, LOESS correction and ln transformation. Green arrows indicate the possible direction of some metabolic processes that took place in patients with TMEM70d and TMEM70d-SCADd.

In univariate analysis, 128 (SCADd), 138 (TMEM70d) 136 (TMEM70d-SCADd) discriminant metabolites were found in comparison with the control group (Supplementary Table S3). In addition, results of comparisons between patient groups can also be found in this table. Boxplots of the most significant urinary metabolites are presented in Fig. 2D. Intermediates of butyryl-CoA metabolism (butyrylcarnitine, butyrylglycine, EMA and MSA) were accumulated in the urine of SCADd and TMEM70d-SCADd patients. 3-Methylglutaconate (3MGC), 2-methylglutaconate (2MGC), 3-methylglutarate (3MG), 3-methylglutarylcarnitine, 3-hydroxy-3-methylglutarate, metabolites from branched-chain amino acid degradation as well as pyruvate, lactate, alanine and citric acid cycle intermediates (citrate, isocitrate, aconitate, 2-oxoglutarate, succinate, fumarate and malate) were significantly elevated in the urine of TMEM70d and TMEM70d-SCADd patients. The carnitine and glycine conjugates acetylcarnitine, propionylcarnitine, tiglylcarnitine and tiglylglycine were increased in these deficiency groups in comparison with the control group. Changes in metabolites were graphically visualized in the metabolic map (Fig. 3), which shows the relationship between metabolites in the respective metabolic pathways.

Within the group of SCADd patients, one symptomatic patient was clinically different. However, his metabolic/lipidomic profile was similar to the others in the group (data not shown).

### 3.2 Serum targeted metabolomics

**Fig. 4:**
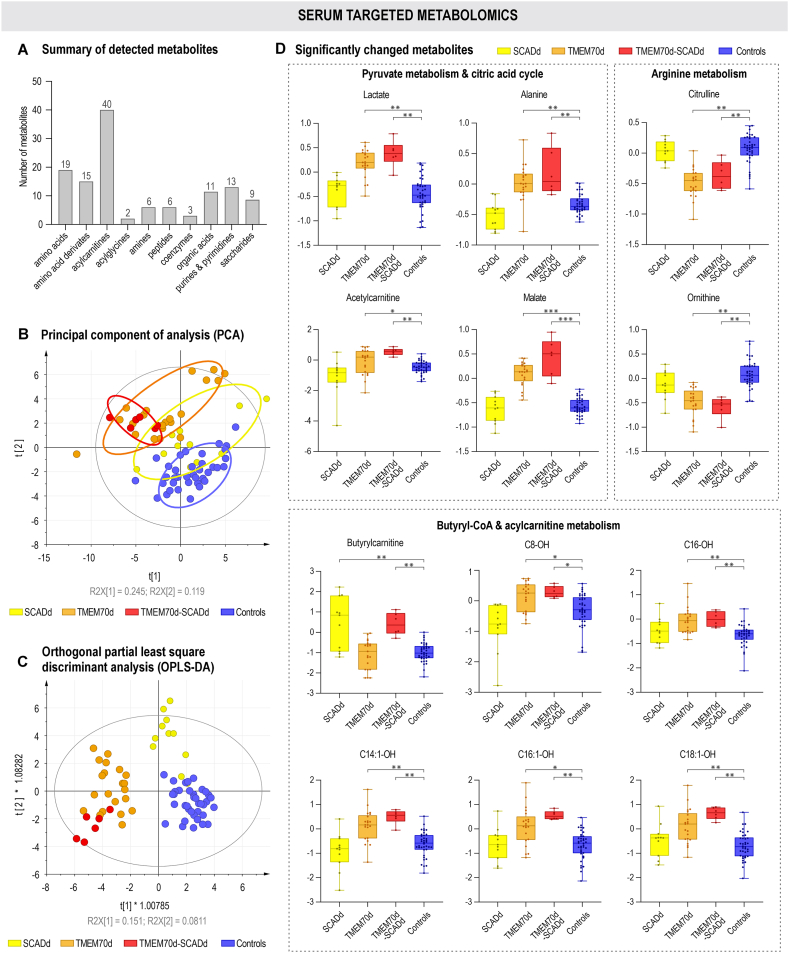
Serum metabolomics. The number of metabolites detected within groups in serum samples (A). Multivariate statistical analysis (B and C) unsupervised PCA and supervised OPLS-DA. Colour-coded sample groups: SCADd (yellow), TMEM70d (orange), TMEM70d-SCADd (red) patients and control subjects (blue). Summary of the most significantly changed ones (D). Statistical significance was assessed by p-values after Benjamini-Hochberg correction (∗<0.05, ∗∗<0.01, ∗∗∗<0.001), the y-axis represents the relative abundance after the application of internal standards, LOESS correction and ln transformation.

Serum targeted metabolomic analysis detected 124 metabolites (Fig. 4A). Similar to urine metabolomics, all studied groups were mostly separated from controls in both multivariate analyses. In PCA, TMEM70d and TMEM70d-SCADd patients were more distant from controls compared with SCADd. OPLS-DA scatter plot almost completely clustered the studied groups. Compared to urine metabolomics, where TMEM70d-SCADd grouped closer to SCADd, the serum metabolic profile of TMEM70d-SCADd appears to be more similar to the TMEM70d in OPLS-DA analysis (Fig. 4B and C). Permutation analysis evaluated the accuracy (R2Y = 0.985/0.976/0.981) and predictive ability (Q2 = 0.782/0.922/0.870) for the SCADd/TMEM70d/TMEM70d-SCADd compared to control group (Supplementary Fig. S1B).

**Fig. 5:**
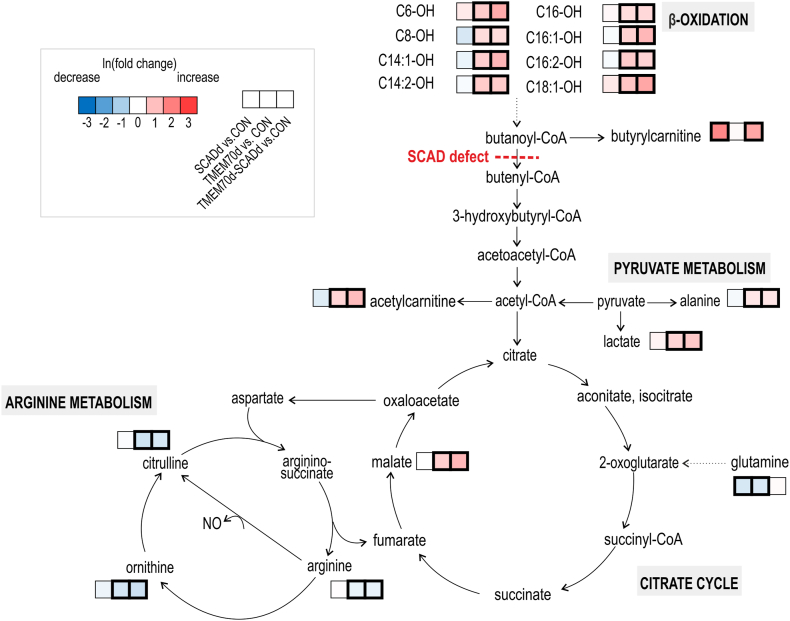
Metabolic map summarizing alterations in serum metabolites in SCADd, TMEM70d and TMEM70d-SCADd patients compared to controls (CON). Significant changes in metabolites after Benjamini-Hochberg correction are highlighted by bold border. The used data were treated with internal standards, LOESS correction and ln transformation.

In univariate analysis, statistically significant changes in 14 (SCADd), 56 (TMEM70d) and 51 (TMEM70d-SCADd) metabolites were detected in serum samples when compared with controls (Fig. 4D–Supplementary Table S3). In addition, results of comparisons between patient groups can also be found in this table. Lactate, alanine, acetylcarnitine (pyruvate metabolism) and malate (citric acid cycle) were significantly elevated in TMEM70d and TMEM70d-SCADd patients compared with controls. In addition, accumulation of hydroxylated acylcarnitines with short-, medium- (C4-OH, C6-OH, C8-OH) and long-chain (C14:1-OH, C14:2-OH, C16-OH, C16:1-OH, C16:2-OH and C18:1-OH) and in contrast decreased glutamine, ornithine, citrulline and arginine (arginine metabolism) were observed in TMEM70d and TMEM70d-SCADd groups. Butyrylcarnitine was highly elevated in the serum of SCADd and TMEM70d-SCADd individuals. All the above significant changes in serum are summarized in the metabolic map (Fig. 5), which shows the relationship between metabolites in the respective metabolic pathways in SCADd, TMEM70d and TMEM70d-SCADd patients.

### 3.3 Serum targeted lipidomics

**Fig. 6:**
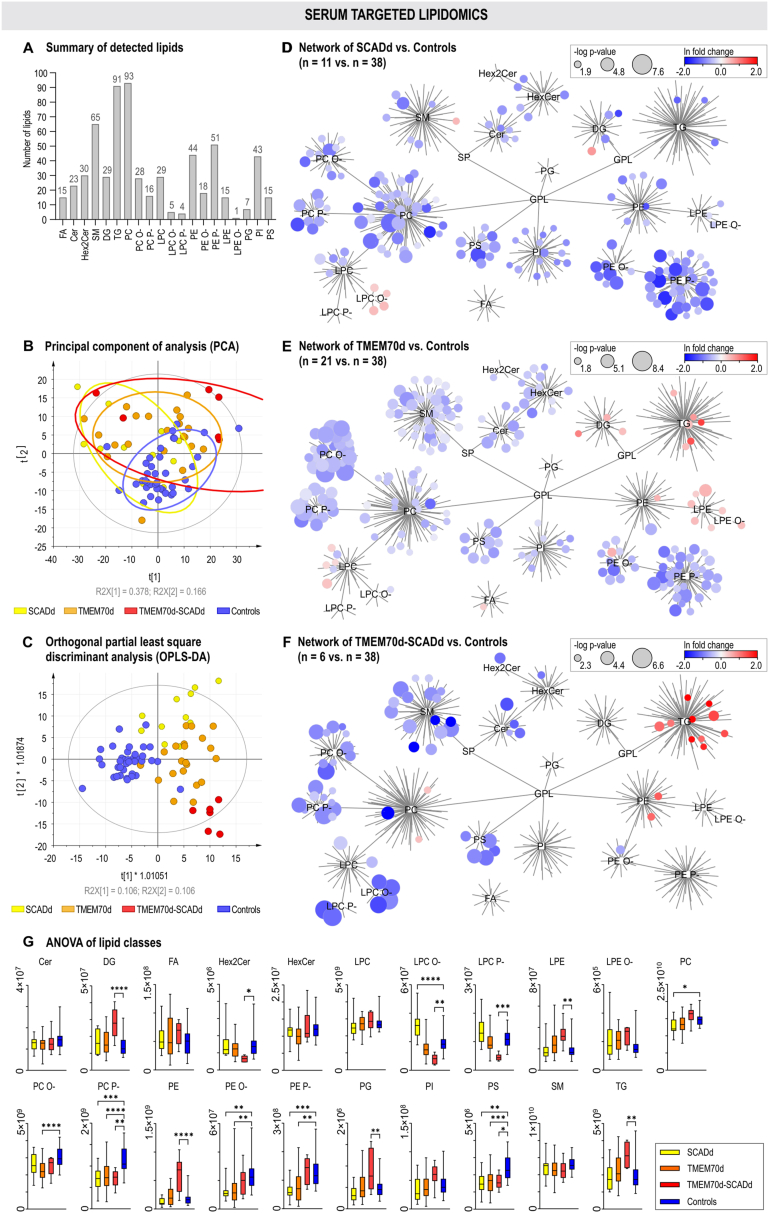
Serum lipidomics. The number of detected lipids within classes in serum samples (A). Multivariate statistical analysis (B and C) unsupervised principal component analysis (PCA) and supervised orthogonal partial least square discriminant analysis (OPLS-DA). Colour-coded sample groups: SCADd (yellow), TMEM70d (orange), TMEM70d-SCADd (red) patients and control subjects (blue). Visualisation of the lipidome of SCADd (D), TMEM70d (E) and TMEM70d-SCADd (F) patients versus controls. The network is constructed as individual lipids connected to their lipid classes: ceramides (Cer), hexosylceramides (HexCer), dihexosylceramides (Hex2Cer), lysophosphatidylcholines (LPC), lysophosphatidylcholine plasmalogens/plasmanyls (LPC P-/O-), phosphatidylcholines (PC); phosphatidylcholine plasmalogens/plasmanyls (PC P-/O-), lysophosphatidylethanolamines (LPE), lysophosphatidylethanolamine plasmanyls (LPE O-), phosphatidylethanolamines (PE), phosphatidylethanolamine plasmalogens/plasmanyls (PE P-/O-), phosphatidylinositols (PI); phosphatidylserines (PS), phosphatidylglycerols (PG), sphingomyelins (SM); diacylglycerols (DG), triacylglycerols (TG) and free fatty acids (FA). The size of nodes corresponds to the -log p-value of a *t*-test and colour coding to the difference of medians (ln transformed). Analysis of variance of lipid classes as sum of intensities for all lipids in each class (y-axis); p-value of ∗ < 0.05, ∗∗ < 0.01, ∗∗∗ <0.001, ∗∗∗∗ <0.0001 (G).

Using the targeted lipidomics method, 622 lipids belonging to 21 lipid classes and subclasses representing ceramides (Cer), hexosylceramides (HexCer), dihexosylceramides (Hex2Cer), lysophosphatidylcholines (LPC), lysophosphatidylcholine plasmalogens/plasmanyls (LPC P-/O-), phosphatidylcholines (PC); phosphatidylcholine plasmalogens/plasmanyls (PC P-/O-), lysophosphatidylethanolamines (LPE), lysophosphatidylethanolamine plasmanyls (LPE O-), phosphatidylethanolamines (PE), phosphatidylethanolamine plasmalogens/plasmanyls (PE P-/O-), phosphatidylinositols (PI); phosphatidylserines (PS), phosphatidylglycerols (PG), sphingomyelins (SM); diacylglycerols (DG), triacylglycerols (TG) and free fatty acids (FA) were identified in the serum samples (Fig. 6A). PCA was used for a general overview of the variability of the data, where clustering of the control samples and separation of most of the patient samples apart from controls was observed on the score plot (Fig. 6B). Although no separation of the patient subgroups themselves was observed in the PCA score plot, using OPLS-DA these groups were separated from controls and each other (Fig. 6C). The original OPLS-DA model was validated by permutation evaluation, achieving accuracy (R2Y = 0.826/0.922/0.986) and predictive ability (Q2 = 0.438/0.710/0.822) for SCADd/TMEM70d/TMEM70d-SCADd compared to control group (Supplementary Fig. S1C).

Furthermore, we focused on comparing differences in serum lipids using univariate statistics and visualisation of systematic changes using lipid networks and boxplots of summed lipids divided into lipid classes (Fig. 6D–G). Common trends of all patient groups compared to controls were represented by significantly decreased levels of glycerophospholipids (mainly PC O-/P-, PE O-/P-, PS) such as PC P-36:0, PE P-18:0/20:5 and PS 18:0_22:6 and sphingolipids (SM and Cer) such as SM 36:2, SM 40:4, Cer d18:2/22:2 and Cer d18:1/22:2. When we focused on specific changes in SCADd patients compared to controls, we found significantly increased levels of LPC O- (e.g. LPC O-20:1 and LPC O-18:0) and decreased levels of polyunsaturated LPC (e.g. LPC 20:5 and LPC 22:6) and PI (e.g. PI 16:0_22:6 and PI 20:0_20:4). In the group of TMEM70d patients compared to controls we found increased levels of LPE and LPE O- (e.g. LPE 20:2 and LPE O-18:3), DG and TG (e.g. DG 16:0_18:2 and TG 54:4) and decreased PI (e.g. PI 18:0_22:5). Lastly, when comparing TMEM70d-SCADd versus controls we found decreased levels of LPC P- (e.g. LPC P-16:0 and LPC P-18:0) and LPC O- (e.g. LPC O-20:1 and LPC O-18:0) and increased levels of TG (e.g. TG 56:4 and TG 56:3) and PE (e.g. PE 18:1_18:1 and PE 38:2).

### 3.4 ROC analysis

The diagnostic value and clinical utility of all statistically significant metabolites and lipids were evaluated using ROC analysis (Supplementary Table S4). Analytes with AUC >0.9 are summarized in the text below. In serum metabolomics, ethylmalonate, nonanoylcarnitine and methionine were evaluated for SCADd. The ROC analysis showed a higher diagnostic power for TMEM70d (malate, nonanoylcarnitine, citrulline, ornithine, lactate and 3-hydroxybutyrylcarnitine) and especially for TMEM70d-SCADd (total of 25 analytes). Compared to serum metabolomics, a higher number of analytes were obtained in urine in all patient groups (8 for SCADd, 15 for TMEM70d and 91 for TMEM70d-SCADd). In serum lipidomics, 15 lipids were found in SCADd, mostly from the PC and PE classes, whereas only 5 lipids from the PC class were found in TMEM70d. Similar to urine, the highest number of lipids (n = 41) was observed in TMEM70d-SCADd, mainly from the PC, LPC and SM lipid classes.

## 4 Discussion

### 4.1 Overview of pathobiochemical interconnections

**Fig. 7:**
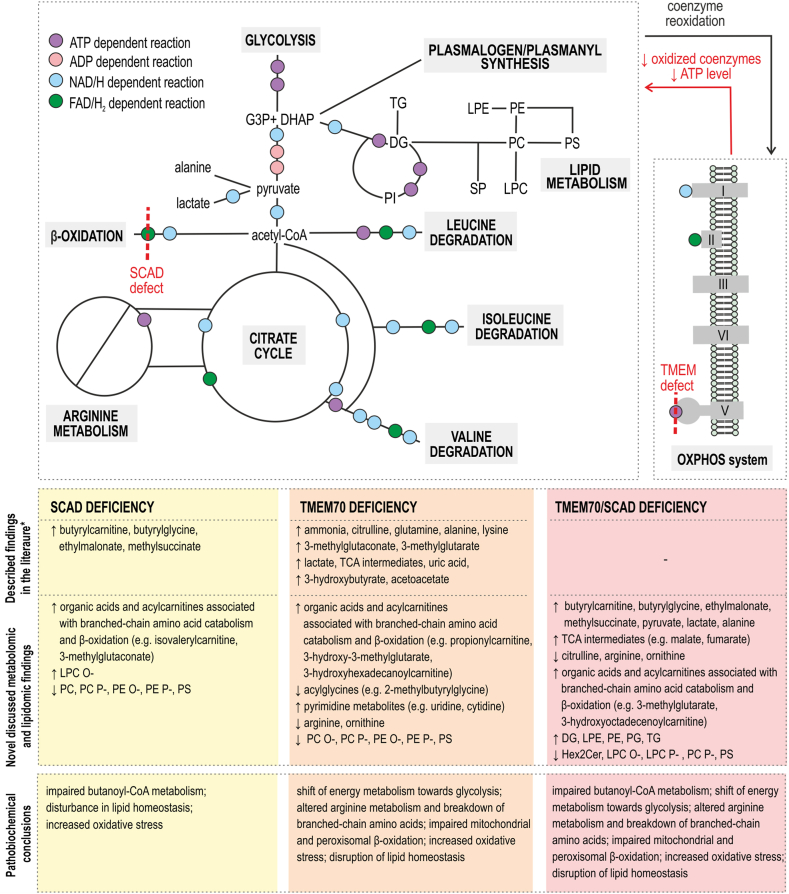
Overview of metabolic pathways with schematic representation of ATP-, ADP-, NAD^+^- and FAD-dependent reactions and depiction of the SCAD and ATP synthase block. Known biochemical findings for SCADd, TMEM70d and TMEM70d-SCADd from the literature [∗12, 28, 29] and novel metabolic and lipidomic changes discussed in detail below are highlighted. Glyceraldehyde-3-phosphate (G3P), dihydroxyacetone phosphate (DHAP), dihexosylceramides (Hex2Cer), lysophosphatidylcholines (LPC), lysophosphatidylcholine plasmalogens/plasmanyls (LPC P-/O-), phosphatidylcholines (PC); phosphatidylcholine plasmalogens/plasmanyls (PC P-/O-), lysophosphatidylethanolamines (LPE), phosphatidylethanolamines (PE), phosphatidylethanolamine plasmalogens/plasmanyls (PE P-/O-), phosphatidylinositols (PI); phosphatidylserines (PS), phosphatidylglycerols (PG), sphingolipids (SP); diacylglycerols (DG), triacylglycerols (TG) and free fatty acids (FA).

Mitochondrial disorders affect a number of metabolic pathways due to the central role of mitochondria in cellular metabolism. The disruption of TMEM70 results in impaired ATP synthase function, leading to a reduction in mitochondrial ATP production and an imbalance in the cellular redox state. Alterations in TMEM70 influence NAD^+^/FAD-dependent enzymatic reactions and ATP-requiring processes (Fig. 7). SCADd impairs the initial step of short-chain fatty acid β-oxidation, resulting in the accumulation of butyryl-CoA and its derivatives, EMA and MSA [12]. These metabolites disrupt several biochemical processes; for example, EMA inhibits the electron transport chain and creatine kinase activity [27], exacerbating mitochondrial dysfunction and oxidative stress. When TMEM70 and SCAD deficiencies coexist in patients, the combined effects on metabolism are synergistic. The accumulation of metabolites from both defects amplifies the inhibition of critical pathways.

Increased NADH/NAD^+^ and FADH_2_/FAD ratios inhibit citric acid cycle enzymes and β-oxidation, shifting energy metabolism towards glycolysis. The resulting accumulation of pyruvate is converted to lactate and alanine, typical markers of mitochondrial disorders [30]. Despite the inhibition of the citric acid cycle, Chen et al. described an anaplerotic reductive glutamine flux in cells with severe OXPHOS deficiency (>55 % inhibition of mitochondrial respiration). Glutamine-derived 2-oxoglutarate is carboxylated to isocitrate and converted to citrate, providing acetyl-CoA for biosynthetic processes [31]. This mechanism, together with increased amino acid catabolism, may explain the increased acetylcarnitine levels in TMEM70d and TMEM70d-SCADd patients, as β-oxidation and pyruvate dehydrogenase activity are presumably inhibited by the reduced availability of oxidized coenzymes [30,32,33]. Furthermore, our data demonstrate a decrease in serum glutamine levels in all patients except the TMEM70d-SCADd group. In individuals with SCADd, glutamine anaplerosis may serve as a compensatory mechanism for the impaired supply of acetyl-CoA, resulting from incomplete β-oxidation.

Elevated levels of hydroxylated long-chain acylcarnitines were observed in the serum of TMEM70d and TMEM70d-SCADd patients. Mitochondrial β-oxidation, the main pathway for fatty acid degradation, depends on oxidized coenzymes and ATP availability for the initial fatty acid activation. β-Oxidation also takes place in peroxisomes where predominantly long-chain, very long-chain, dicarboxylic and unsaturated fatty acids are catabolized [34]. The initial step of peroxisomal β-oxidation is catalysed by FAD-dependent acyl-CoA oxidase (AOX, EC 1.3.3.6) to form enoyl-CoAs. The reoxidation of FADH_2_ does not proceed through the respiratory chain; electrons from the reaction are directly transferred to molecular oxygen to form H_2_O_2_ [35]. Subsequently, enoyl-CoAs are converted into 3-hydroxyacyl-CoAs, which undergo NAD^+^-dependent dehydrogenation. Until this reaction is probably reduced in patients with OXPHOS defects. This is consistent with reports of increased hydroxylated acylcarnitines in patients with mitochondrial myopathy, including individuals with reduced respiratory chain complexes activity [36]. The observed increase in shorter hydroxylated acylcarnitines in TMEM70d and TMEM70d-SCADd patients may be linked to amino acid degradation [30,37]. Reduced serum levels of C9 carnitine, consistent with our findings, have been reported in long-chain 3-hydroxyacyl-CoA dehydrogenase deficiency, mitochondrial trifunctional protein deficiency and Leigh syndrome, presumably due to impaired breakdown of branched-chain or odd-chain fatty acids [[38], [39], [40]].

A number of significant changes in the metabolism of leucine, isoleucine, and valine were observed in patients with TMEM70d and TMEM70d-SCADd. Increased levels of 3MG and 3MGC, which have been previously reported in TMEM70d [28] and other mitochondrial diseases such as Barth syndrome [41,42], may result from the diversion of acetyl-CoA into 3MGC synthesis instead of the inhibited citric acid cycle. The formation of 3MGC involves four enzymatic steps in leucine metabolism, followed by its reduction and hydrolysis to 3MG, which is excreted in the urine [42,43]. The origin of 2MGC remains unclear, although its elevation has been associated with beta-ketothiolase deficiency, propionic acidemia, and methylmalonic acidemia [44]. In these conditions, 2MGC is thought to be the product of 3-methylcrotonyl-CoA carboxylase activity (EC 6.4.1.4). Our findings demonstrate a decrease in urinary excretion of isovalerylglycine, 2-methylbutyrylglycine and isobutyrylglycine in patients with TMEM70d and TMEM70d-SCADd. The underlying mechanism remains to be elucidated, but one hypothesis is a possible reduction in the availability of glycine for mitochondrial acylglycine synthesis. A decrease in glycine levels and an increase in serine levels, its precursor, were observed in the serum of patients with TMEM70d and TMEM70d-SCADd (Supplementary Table S3). It has been reported that mitochondrial dysfunction leads to an increase in serine biosynthesis and impaired NAD(P)^+^ -dependent mitochondrial production of formate, during which glycine is formed [45].

Serum metabolomics revealed reduced citrulline, arginine, and ornithine in TMEM70d and TMEM70d-SCADd patients. Hypocitrullinemia has been reported in several mitochondrial diseases, including mitochondrial encephalomyopathy, lactic acidosis, and stroke-like episodes syndrome (MELAS); neurogenic weakness, ataxia and retinitis pigmentosa (NARP) syndrome; Leigh syndrome and Pearson syndrome [[46], [47], [48], [49]], though hypercitrullinemia has been observed during metabolic crises in TMEM70d patients [28,29]. The majority of circulating citrulline is produced by mitochondrial glutamine conversion in enterocytes. Its primary utilisation is in the kidney, where it serves as a precursor for arginine biosynthesis [[50], [51], [52]]. The mechanism of hypocitrullinemia in mitochondrial disorders remains unclear but may involve reduced ATP availability for enzymes like carbamoyl phosphate synthetase 1 (CPS1, EC 6.3.4.16) [47]. Other potential explanations include increased urinary citrulline excretion or its diversion to orotate synthesis in pyrimidine metabolism, as previously discussed in the context of Pearson syndrome [49]. The present study revealed urinary accumulation of citrulline and arginine in TMEM70d-SCADd patients, while no significant changes were detected in TMEM70d patients compared to controls. Elevated urinary orotate and pyrimidine metabolites (uridine, cytidine) were observed in both OXPHOS-deficient groups. The reduced availability of glutamine for citrulline formation may also play a role, consistent with reports of hypercitrullinemia in patients with elevated glutamine levels [28,29]. However, our results showed a decrease in serum glutamine levels only in TMEM70d patients. Further studies are needed to elucidate the mechanisms underlying hypo- and hypercitrullinemia in TMEM70d patients. One consequence of reduced citrulline and arginine levels is impaired nitric oxide (NO) synthesis, reported in some mitochondrial diseases [53]. NO, produced from arginine by endothelial NO synthase, is crucial for vascular smooth muscle relaxation, maintaining microvascular patency and blood flow [54]. Altered NO synthesis may contribute to pulmonary arterial hypertension, a manifestation observed in newborns with TMEM70d [55,56].

In addition to the characterized changes in central metabolism revealed by metabolomics, the pathobiochemical changes in TMEM70d and SCADd patients were also related to alterations in lipid levels. Regarding the general decrease of PS in all patient groups, this can be associated with phosphatidylserine synthase (PTDSS, EC 2.7.8.29) which regulates cell growth, lipid storage and mitochondrial function [57]. Dysregulation of PTDSS has been linked with a metabolic shift from phospholipid synthesis to neutral lipid synthesis and their ectopic accumulation and impairment of mitochondrial protein import and aberrant mitochondrial morphology [57]. Dysregulation of PTDSS is associated with the aforementioned accumulation of neutral lipids and it has been discovered previously that the elevated TG (as we observed in TMEM70d and especially in TMEM70d-SCADd patients compared to controls) are related to mitochondrial dysfunction [58] and that accumulation of TG activates the mitochondrial apoptosis pathways in many cell types [59]. However, TG are primarily associated with energy storage and transport, and their connection with mitochondrial dysfunction and PTDSS regulation has not yet been fully elucidated. Although SCADd is also partially attributed to an energy imbalance, oxidative stress is much more discussed [12]. EMA, a hallmark of SCADd, inhibits creatine kinase activity in rats (cerebral cortex and skeletal muscle), causing reduced glutathione levels, increased lipid peroxidation, protein oxidation [60,61] and additionally, inhibition of mitochondrial electron transport chain activity (in human skeletal muscle in vitro) [27]. Based on many studies reviewed elsewhere [12], we can conclude that SCADd cells exhibit chronic oxidative stress. Similarly, a very close group of IMD presented by deficiency of medium-chain acyl-CoA dehydrogenase showed elevated levels of oxidized phospholipids as indicators of increased oxidative stress [62]. Concerning increased oxidative stress, our findings of decreased levels of plasmalogen lipids (PE P- and PC P-, LPC P-) in SCADd, TMEM70d, and TMEM70d-SCADd patients compared to controls are of particular interest. PC P-, PE P- and LPC P- contain a vinyl ether bond in their structure capable of scavenging free oxygen, peroxide and other radicals [63]. Furthermore, PE P- species have been previously described as potential antioxidants due to their protective effect during oxidative stress [64]. The exact mechanism of the decrease of PE P- is still unknown but considering the current literature, it appears to be a combination of not only oxidative stress but also inflammation [65], membrane lipid raft remodeling and peroxisome dysfunction [66]. Generally, the decreased levels of plasmalogens can be explained by the reduced capacity for normal plasmalogen biosynthesis and remodeling caused by impaired mitochondrial OXPHOS and fatty acid oxidation, reduced ATP generation and increased oxidative stress and potential redox disturbances occurring in SCADd and TMEM70d [27,57,61,62].

### 4.2 Synergy metabolic effects in patients with TMEM70d-SCADd

This study provided a unique opportunity to investigate the synergy effect in TMEM70d-SCADd patients caused by the combination of two different defects, SCADd and TMEM70d. In urine as well as serum, synergistically increased excretion and levels of citrate, 2-oxoglutarate, succinate, fumarate, pyruvate, lactate, acetylcarnitine, and hydroxylated acylcarnitines were observed (Fig. 4). Additionally, synergistically increased polyunsaturated TG and PE and decreased polyunsaturated SM (interestingly, as in both PE and SM with seven double bonds), Cer, and PC O-/P- were found (Fig. 6, Supplementary Table S3). Despite the lower power of the study (due to the small sample size), the increased accumulation of these metabolites and lipids compared to TMEM70d can be associated with an effect on enhanced suppression of β-oxidation, citrate cycle and activation of pyruvate metabolism and oxidative stress (due to impaired oxidative phosphorylation).

### 4.3 Metabolomics and lipidomics in diagnosing of SCADd, TMEM70d and TMEM70d-SCADd

The strength of metabolomics and lipidomics lies in comprehensive information about metabolites and lipids. Supervised multivariate statistical tools have been successfully applied to these data to achieve better clinical efficiency compared to classical univariate methods. In the case of SCADd, well-known markers (such as butyrylcarnitine) show unsatisfactory sensitivity and specificity values. Multivariate discriminant analysis (OPLS-DA) using metabolomic/lipidomic data is proposed as a significantly more efficient diagnostic and classification tool. In TMEM70d and TMEM70d-SCADd, we identified for the first time 2-methylglutaconate as a potential novel biomarker, which shows high excretion in the urine of patients. This biomarker does not seem to be entirely unique, as it has previously been found elevated in some selected organic aciduria (e.g., methylmalonic or propionic aciduria [[39], [44]]). However, it does provide complementary information of TMEM70d subclassification. In addition, ROC analysis confirmed the previously mentioned findings of clinically relevant metabolites. Serum metabolomics identified metabolites with high clinical efficacy, especially in TMEM70d-SCADd. In contrast, the diagnosis of SCADd is more uncertain. In urine, the changes are more pronounced. Calculated AUCs of 100 % were found for 2, 2 and 16 metabolites for SCADd, TMEM70d and TMEM70d-SCADd respectively. Among these, urinary butyrylglycine and ethylmalonate, products of the metabolism of accumulated butyryl-CoA, are well established biomarkers of SCAD deficiency [12]. In addition, 3-methylglutaconate and 3-methylglutarate, derived from leucine metabolism, showed 100 % sensitivity and specificity in distinguishing TMEM70-deficient patients from controls, highlighting their diagnostic potential for screening selected mitochondrial diseases [41]. The combined defect remains unexplored in terms of metabolic and lipidomic biomarkers. While studies of mitochondrial diseases in general have identified some of the metabolites we observed [30], the TMEM70d-SCADd group revealed significant metabolites that are found separately in each disease, and in some cases with a synergistic effect. Our work is unique in that it is the first time that serum lipidomics has been studied in TMEM70d and TMEMd-SCADd patients, respectively. Surprisingly, compared to serum metabolomics, serum lipidomics revealed more significant changes in univariate, multivariate and ROC analyses and has the potential to provide a more efficient diagnosis for all the diseases studied. Furthermore, lipidomics appears to be a promising approach even for the well-described SCAD deficiency.

### 4.4 Future outlooks

In the future, the pathobiochemical changes associated with TMEM70d and SCADd (respectively TMEM70d-SCADd), especially arginine metabolism, will need to be further investigated. It will also be beneficial to determine whether the metabolome and lipidome pathologies we found are related to the clinical manifestations of these disorders. Differences in significantly affected analytes can also be expected during the course of the diseases. Monitoring of metabolome and lipidome changes during patient treatment could also be beneficial. Moreover, studying a larger cohort of patients with the combined defect will not only be essential for this purpose but will also confirm the findings of this paper. Our targeted lipidomic method is not able to detect oxidized lipids, as a completely different analytical setup would be required, and it would be advantageous to test new advanced epilipidomic approaches [67] in the future to better characterize oxidative stress in SCADd, TMEM70d and TMEM70d-SCADd patients.

## 5 Limitations of the study

The main limitation of this study was the restricted number of patients with combined TMEM70-SCADd defect, which has not yet been characterized. As a result, the testing and validation phases of the experiments could be carried out. Additionally, limited numbers of patient samples may have affected the statistical significance of the results. Differences in the metabolic profile depending on the type of mutation were not performed due to the small number of patients. The age of the studied patients and healthy subjects was 1–18 years. Acute cases of younger patients, in whom metabolic crisis was assumed, were not included in the study due to the assumption of too dramatic changes that would affect the objectivity of the results obtained. The median age of patients and controls differed slightly. The control group also had a higher proportion of women. Only statistically significant results verified by Benjamini-Hochberg correction were commented. Due to the rarity of the diagnosis, for some patients, several samples were included in the statistical analysis. No statistically significant changes in metabolite/lipid levels were observed in the replicate samples. Furthermore, although our targeted metabolomic and lipidomic methods provide analysis of a large dataset (300+ metabolites and 600+ lipids), it cannot be excluded that an untargeted approach would not detect changes in other unexpected analytes. The metabolome and lipidome of patients and controls may have been influenced by medications, diet and country of origin.

## 6 Conclusion

The study focused on pathobiochemical changes found in the serum and urine of patients with SCADd, TMEM70d and TMEM70d-SCADd by targeted metabolomics and lipidomics. Impaired oxidative phosphorylation was manifested by disruption of central metabolism and imbalance of the citric acid cycle, β-oxidation, degradation of branched-chain amino acids and arginine metabolism in TMEM70d and TMEM70d-SCADd patients. Lipidomic analysis revealed increased oxidative stress due to decreased levels of PE P-/PC P- (which act as antioxidants) and we also found a complex interplay between PS (decreased) and TG (increased) pointing to pathobiochemical effect leading to mitochondrial dysfunction. The metabolic processes discussed in this work may provide missing information necessary to understand the early diagnosis and treatment of these mitochondrial disorders.

## CRediT authorship contribution statement

**Dana Dobešová:** Writing – review & editing, Writing – original draft, Methodology, Data curation, Conceptualization. **Matúš Prídavok:** Writing – original draft, Validation, Methodology, Data curation, Conceptualization. **Radana Brumarová:** Writing – original draft, Visualization, Software, Investigation. **Aleš Kvasnička:** Writing – original draft, Visualization, Methodology, Conceptualization. **Barbora Piskláková:** Writing – original draft, Validation, Methodology, Data curation. **Eliška Ivanovová:** Writing – original draft, Software, Methodology. **Katarína Brennerová:** Writing – original draft, Resources. **Jana Šaligová:** Writing – original draft, Resources. **Ľudmila Potočňáková:** Writing – original draft, Resources. **Simona Drobňaková:** Writing – original draft, Resources. **Jana Potočňáková:** Writing – original draft, Resources. **David Friedecký:** Writing – original draft, Visualization, Validation, Supervision, Resources, Project administration, Investigation, Funding acquisition, Data curation, Conceptualization.

## Ethics approval and consent to participate

The study was approved by the Ethics Committee of The National Institute of Children's Diseases, Bratislava, Slovakia (No. EK6/3/2023). Written informed consent was obtained from all participants. In addition to the consent of the parents/legal guardians, the consent of the minor(s) has been obtained where necessary.

## Data availability

Data confirming the results of this study are available from the corresponding author upon reasonable request. The raw data and datasets generated and/or analyzed in this study are available in the MassIVE repository (ID MSV000094418) and at https://doi.org/doi:10.25345/C5D50G81T.

## Funding

This work was supported by the Agency for Medical Research of the Czech Republic (NU20-08-00367) and Ministry of Health of the Czech Republic (MH CZ - DRO FNOl, 00098892).

## Declaration of competing interest

No potential conflict of interest was reported by all authors.

## Figures and Tables

**figs1:**